# Efficacy of a Computerized Sensor System for Evaluation and Training of Dizzy Patients

**DOI:** 10.3390/s100807602

**Published:** 2010-08-12

**Authors:** Chung-Lan Kao, Wan-Ling Hsieh, Shuu-Jiun Wang, Shih-Jen Chen, Shun-Hwa Wei, Rai-Chi Chan

**Affiliations:** 1 Department of Physical Medicine and Rehabilitation, Taipei Veterans General Hospital, 201 Shih-Pai, Road, Section 2, 11217, Taipei, Taiwan; E-Mails: wlhsieh3@vghtpe.gov.tw (W.-L.H.); rcchan@vghtpe.gov.tw (R.-C.C); 2 School of Medicine, National Yang-Ming University, No. 155, Section 2, Linong Street, Taipei, 11221, Taiwan; E-Mails: sjwang@vghtpe.gov.tw (S-J.W); sjchen@vghtpe.gov.tw (S.-J.C.); 3 Center for Geriatrics & Gerontology, Taipei Veterans General Hospital, 201 Shih-Pai Road, Section 2, 11217, Taipei, Taiwan; 4 Institute of Physical Therapy and Assistive Technology, National Yang-Ming University, No. 155, Section 2, Linong Street, Taipei, 11221, Taiwan; 5 Department of Neurology, Neurological Institute, Taipei Veterans General Hospital, 201 Shih-Pai, Road, Section 2, 11217, Taipei, Taiwan; 6 Department of Ophthalmology, Taipei Veterans General Hospital, 201 Shih-Pai, Road, Section 2, 11217, Taipei, Taiwan

**Keywords:** dizziness, balance, dynamic visual acuity, center of pressure, vestibular hypofunction

## Abstract

Patients with vestibular hypofunction often experience dizziness and unsteadiness while moving their heads. Appropriate sensors can effectively detect a patient’s dynamic visual acuity and associated body balance control. Forty-one vestibular-deficit patients and 10 normal individuals were invited to participate in this study. Questionnaires, clinical assessment scales and objective measures were evaluated on participants’ first visits. After 12 sessions of training, all scales were evaluated again on vestibular-deficit patients. The computerized system was composed of sensors, including a gyro and strain gauges, data acquisition accessories and LabVIEW software. Results revealed that the system could effectively distinguish normal subjects from subjects with vestibular deficits. In addition, after a rehabilitation program, subjects’ subjective and objective performances were significantly improved. Based on our results, we concluded that the present system, which uses a gyro and strain gauges, may provide an effective method for assessing and treating vestibular-deficit patients.

## 1. Introduction

Dizziness is a clinically relevant problem commonly experienced by the general population [1]. The causes of dizziness can be classified into two broad categories; those associated with pathologies intrinsic to the vestibular system, and those extrinsic to pathologies of the vestibular system [2]. Diseases affecting the vestibular system can result in symptoms of imbalance, dizziness and oscillopsia [3]. Disruption of the vestibulo-ocular reflex (VOR), in the absence of adequate compensatory mechanisms or integration with other inputs, leads to a decline in VOR gain [4].

The VOR is a reflex mechanism that occurs during movements of the head. It consists of coordinated movements of the ocular globe that maintain visual stabilization. This reflex helps to reduce retinal slip and ensures visual acuity so that clarity of vision is maintained during head movements. The vestibulo-ocular reflex functions even while the individual remains motionless. Postural sway can induce compensatory movements through the VOR [5]. This compensatory movement of the ocular globe during VOR is very rapid; the duration may be as short as 5–7 ms [6]. Retinal slip of >2 deg/second leads to a significant decline in visual acuity [7]. As the VOR operates within an extremely short time span, it plays a crucial role in maintaining and sustaining visual stabilization.

Patients with vestibular hypofunction often suffer from gaze instability. This is attributed to an inability to maintain visual stabilization during head movements faster than 120 deg/second. In more severe cases, individuals’ balance may be affected, which may cause falls [8]. Dynamic visual acuity (DVA) assessment, measures an individual’s ability to maintain visual stabilization during head movements, and can provide an approximation of VOR function [8]. As for balance function evaluation, clinical and laboratory based research has led to various methods of measuring balance. Of these, the three/single-axis balance plate is the most widely used to evaluate balance. It can accurately and objectively measure sway in an individual’s center of pressure (COP). It also has minimal environmental constraints, making it an ideal measurement tool for monitoring COP displacement. Paloski *et al.* [9] and Clark [10] used posturography to detect the postural sway during head movement. Therefore, the balance plate allows the investigator to detect whether a vestibular deficit patient experiences loss of balance due to dizziness during head motions.

While objective evaluation is valuable in assessing vestibular function, it is also important to pay attention to the subjective sensations of individuals. In Meli’s study, objective and subjective performance evaluations as well as measures of quality of life have demonstrated beneficial effects of vestibular rehabilitation [11]. Prior literature has also pointed out that a patient’s subjective evaluation provides a better indication of his/her quality of life than results obtained via objective measurement criteria [12]. Therefore, measurements of vestibular function should include an assessment of daily function as well as other objective measures. Emotional well-being can be assessed using the Hospital Anxiety and Depression Scale (HADS) [13–15]. Similar scales, that are used frequently in the clinical setting, include the Activities-Specific Balance Confidence Scale (ABC) and the Dizziness Handicap Inventory (DHI) [16,17]. The current study uses the ABC, DHI and HADS as indicators of patients’ perceived balance performance, dizziness and anxiety-depression states after rehabilitation. To objectify our findings, the Dynamic Gait Index (DGI) [12] and the Tinetti fall risk performance scale [18] were also applied to evaluate balance functions of vestibular-deficit subjects. The Timed “Up and Go” (TUG) test was also assessed to determine fall risk [19].

Herdman *et al*. [4] indicated that vestibular exercise can improve dynamic visual acuity and reduce the discomfort induced by oscillopsia. The current standard of practice in vestibular rehabilitation primarily involves muscle strengthening, gait and balance training [2] or specific gaze stabilization exercises, as well as repositioning interventions [20], adaptation exercise [2]. Vertigo habituation exercises [21–23] are also effective in reducing symptoms. Virtual reality (VR) has introduced new insights to the field of rehabilitative science in recent years. For individuals suffering from different severity of dizziness, by manipulating various contexts in the virtual environments, for instance, incorporating virtual driving scenes, patients could attain better functional performance in daily activities [24]. Some studies have suggested that vestibular rehabilitation should not focus solely on balance-related training but should incorporate exercises with an emphasis on repetitive head movements, with gradually increased movement frequency and speed, as well as interchangeable visual and vestibular use [21]. It is therefore important to develop a system that incorporates evaluation in addition to rehabilitation, and has the ability to assess the nature of vestibular hypofunction. Rehabilitation will ideally feature exercise activities relevant to activities performed in daily life.

This study reports and evaluates a system that incorporates both evaluation and training of vestibular functions by computerized methods. The computerized system, comprised of a gyro sensor and a balance plate, was developed to detect subjects’ balance ability over a range of head velocities. This device can simultaneously evaluate subjects’ DVA and COP. Unlike most DVA measurement protocols, which assess patients while they sit, our computerized system allows patients to stand while evaluating DVA and COP. This simulates the real movements occurring during daily activities. In this study, we compared a range of different visual acuities and COP displacements while performing a DVA test in healthy individuals, individuals with unilateral vestibular hypofunction (UVH) and individuals with bilateral vestibular hypofunction (BVH). Furthermore, this study compared measures of life quality and disability before and after vestibular rehabilitation.

## 2. Experimental Section

### 2.1. Participants

Patients with unilateral or bilateral vestibular hypofunction were referred from clinic. Healthy volunteers, without a history of dizziness or vertigo, were recruited. The diagnosis of vestibular hypofunction was based on the results of a head thrust test [2], a horizontal head shaking test [2], a and a caloric test [2]. In our laboratory, we use air as the irrigation media in caloric tests (AIRSTAR, Micromedical Technologies, Illinois, USA). The value of slow phase eye velocity (SPEV) was calculated after each irrigation. For UVH, a canal paresis (CP) and directional preponderance (DP) greater than 25% was considered significant. Bilateral vestibular dysfunction was defined as low SPEV (<5 degrees/s). All participants were asked to sign informed consents that had been approved by the Taipei Veterans General Hospital Institutional Review Board. Exclusion criteria were benign paroxysmal positional vertigo confirmed by a positive Hallpike-Dix test, post-traumatic vertigo, degenerative neurological disease, whiplash injury and cognitive impairment.

### 2.2. Equipment and Devices

**Figure 1.**
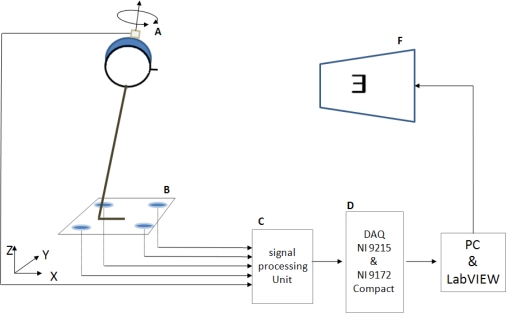
Experiment setup: (A) gyro sensor, (B) balance plate with four strain gauges installed on the four corners of the plate, (C) processing unit for conditioning strain gauge and gyro signals, (D) analog to digital converter and data via through USB cable to PC, (F) the optotype “E” displayed on the monitor that is controlled by the program.

In order to simultaneously measure head rotational speed and body sway, a gyro sensor (CRS03, Silicon Sensing, UK) and balance assessment system (Accurate MSD04, Taiwan) were used. The gyro sensor was fastened to subjects’ heads for detection of head velocities in horizontal and vertical planes. The balance assessment system provided four analog output signals. These four signals were from strain gauges installed on the bottom of the balance plate. All the analog signals were simultaneously sent into NI Compact DAQ 9172 (National Instruments) for performing analog to digital conversion. All sampling rates were 50 Hz. Using strain gauge signals and dimension specification, COP could easily be calculated. LabVIEW 8.5 was used for developing the analysis program. The experimental setup is shown in Figure 1.

### 2.3. SVA and DVA Measurements

Static visual acuity (SVA) was tested while subjects stood on the balance plate 2 m in front of the computer screen. The horizontal level of the monitor was adjusted according to each subject’s visual height. A single optotype “E” was displayed on the monitor. The direction of “E” was altered randomly with an interval of 2 seconds by a computer generated program. The letter size decreased in each acuity line with the interval equivalent to 0.1 LogMAR. The converter of optotypes was equivalent to the Early Treatment Diabetic Retinopathy Study (ETDRS) visual acuity chart. Subjects were tested with their best corrected vision. They were required to indentify the orientation of five optotypes in each acuity line. COP was simultaneously recorded. The test was terminated when a subject failed to identify all five letters on the same acuity line.

DVA tests comprised of two parts, horizontal DVA (hDVA) and vertical DVA (vDVA). Subjects were instructed to oscillate their heads in yaw and pitch planes. The “E” appeared on the monitor only when the speed of subject’s head rotation was between 120 deg/second and 180 deg/second, and the letter “E” stayed on the screen for 85 ms with the interval of 2 seconds. Subjects were asked to stand on the balance plate and indentify the direction of optotypes during head rotation. In order to avoid visual compensation, the presentation of the optotype was controlled by the direction of head movement during vDVA and hDVA. For hDVA tests, the first trial was driven by leftward head movement only and the following trial was driven by rightward head movement only. The same control was applied to the vDVA tests. Each subject was blinded for this control and was required to complete all trials (left, right, up and down). When a subject was unable to identify the letter direction, the system recorded the missed opototypes. The DVA test was terminated when subjects could not identify all five optotypes on the same acuity level.

### 2.4. Data Reduction

To reduce the effect of individual visual acuity differences, the value of hDVA/vDVA was defined as the total number of missed optotypes in the hDVA and/vDVA test minus the number of missed optotypes in the SVA test. This was converted to decimal visual acuity LogMAR ([log1/numerator/denominator]) [8].

In order to evaluate subjects’ balance control abilities while performing DVA tests, COP displacement was recorded only when head rotation velocity was between 120 deg/second and 180 deg/s. Total COP displacement is commonly used for evaluating balance ability [25,26]. In general, smaller total COP displacement at a given time suggests that a subject has a greater ability to maintain stability. On the contrary, a larger total COP displacement suggests that a subject has a lesser ability to maintain stability [27–29]. Nine volunteers (three normal, three UVH and three BVH) were tested twice within one week to determine the test-retest reliability of the system. Results showed that ICC was 0.982 and 0.962 in DVA and COP, respectively. This indicated that the measurements were repeatable.

### 2.5. Clinical Assessment of Outcome Effects

In order to validate the functional improvement in selected daily activities that were associated with DVA training, the present study used several clinical assessment methods. All of these methods have been validated previously [12,16,18,19,30–32]. These methods were DHI [16], ABC [30], HADS [31], visual analogue scale (VAS), Tinetti fall risk performance scale [18], DGI [12] and TUG [19].

#### 2.5.1. Dizziness Handicap Inventory (DHI)

The DHI [16] is a validated 25-item questionnaire to evaluate the functional, emotional and physical problems developed due to dizziness. A higher score on the DHI indicates a greater level of handicap.

#### 2.5.2. The Activities-specific Balance Confidence Scale (ABC)

The ABC scale [30] was developed to assess balance confidence during performance of 16 activities of daily living. A score of 100% indicates full confidence. The average of these 16 items was recorded to reflect the subjective feeling of dizziness.

#### 2.5.3. The Hospital Anxiety and Depression Score (HADS)

The HADS [31] is a self-assessment questionnaire with 14 items (seven for depression and seven for anxiety). Each item contains a 4-point scale question. The HADS measures the psychological state of a patient. A higher score indicates a poorer mental health. The Chinese version of the HADS is consistent with the English version [32].

#### 2.5.4. Visual Analog Scale (VAS)

Subjects were asked to rate the severity of their dizziness from 0 to 10. A higher score indicates greater severity. Visual analog scale is an indicator of patient-centered compliance, which reflects individual patient’s self-perceived sense of discomfort induced by dizziness, vertigo or oscillopsia.

#### 2.5.5 Tinetti Fall Risk Performance Scale (TFRPS)

This scale is based on a series of balance tests ratings from 0 to 2, with 0 indicating severe impairment and 2 indicating normal ability. The maximum total score is 28. A score greater than 25 indicates low risk of falling, and scores less than 19 indicates high risk of falling [18].

#### 2.5.6. Dynamic Gait Index (DGI)

The eight items of the DGI include walking while changing speed and turning the head, walking over and around obstacles, and stair climbing. Scoring of the DGI is based on a 4-point scale. The maximum total score is 24, and scores greater than 19 indicate a low risk for falling [12].

#### 2.5.7. Timed “Up and Go” Test (TUG)

This measure reports the time that it takes a subject to standing from a chair with armrests, walking three meters at a preferred speed, turn around, walking back to the chair and sitting down. Subjects could use assistive devices, but could not receive assistance from other people. For patients with vestibular hypofunction, the cut-off value to indicate a risk for falls is 11.1 seconds [19].

**Figure 2.**
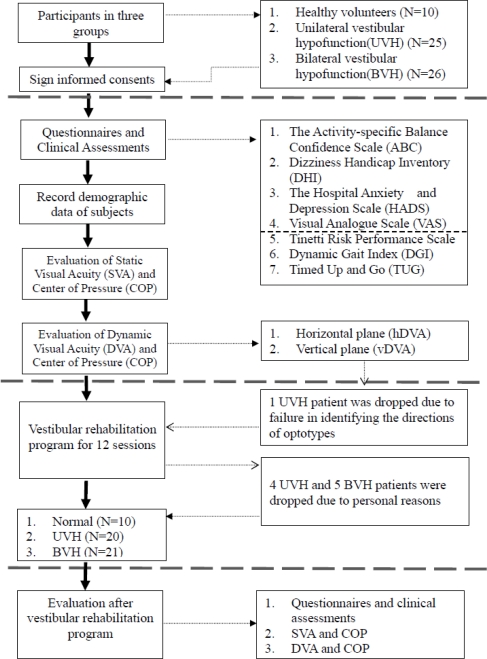
Flow chart of this study.

All subjects were asked to receive clinical assessments before and after completion of the rehabilitation protocol. The flow chart of this study with numbers of patients in each stage is shown in Figure 2.

### 2.6. Rehabilitation Program

We designed a 4-week rehabilitation program (twelve 30-minute sessions). After a preliminary evaluation, participants with vestibular-deficits entered this training program. In each training session (see Table 1), auditory cues of 2 Hz were given at the beginning, with gradual increment to 3 Hz according to individuals’ abilities. We considered subjects’ visual fields to be ±60 degrees. Therefore, peak to peak amplitude of head rotation was 120 degrees. The time for each rotation cycle was 1/6 s. In order to guide the subjects’ head rotations, we used a computer program to provide an auditory beep at a constant time interval of 1/6 seconds.

A gyro was fastened to subjects’ heads for detection of head velocities in horizontal and vertical planes. Patients were asked to rotate their heads and stand on foam in either a tandem stance or while stepping in place. The durations and repetitions of each exercise were dependent upon individual patient’s abilities. Participants were asked to identify and name items in photos appearing on a monitor located 2 m in front of them while moving their heads according to the auditory cues, with a head velocity of 120 to 180 degrees/second. When head velocity was not in the accurate speed range photos would not be shown on the screen. The photos that appeared on the monitor mimicked the environment in our daily life. For example, patients were asked to read the route numbers of buses while stepping and moving their heads, or to name and search for fruits and vegetables in a grocery store. The protocol for rehabilitation training is listed in Table 1.

All clinical assessments were evaluated again after 12 sessions of training. All clinical assessments (pre-training and post-training) were done by the same examiner (W-L Hsieh) who was blinded to the diagnosis of our patients.

### 2.7. Statistical Analysis

The differences in DVA and COP in horizontal/vertical planes among the three groups were compared by one-way ANOVA. Bonferroni *post hoc* analysis was applied to compare the differences between groups. The differences in DVA, COP displacement, HADS, DHI, ABC, VAS, DGI, TFRPS and TUG before and after training were compared by paired-*t* test. Due to multiple comparisons, a p-value of 0.006 or less was considered statistically significant based on bonferroni correction.

## 3. Results and Discussion

### 3.1. Subjects Characteristics

A total of 51 subjects (mean age: 60.71 ± 19.91 years old, 28 males and 23 females) were included in this study. None of the subjects experienced any adverse side-effects such as vomiting or falling during the course of this study. Subjects were categorized as 10 normal individuals, 20 UVH and 21 BVH patients. Normal subjects differed in age (42.4 ± 29.73 years) with other groups (UVH: 69.5 ± 8.19, BVH: 64.6 ± 12.53 years). No difference was observed in gender, static visual acuity (SVA) scores (with best corrected vision) and static COP displacement across groups. (ANOVA, p > 0.1)

### 3.2. Dynamic Visual Acuity and COP Differences between Normal and Vestibular Deficits Individuals

Using the new instrument, we found differences in the mean values of LogMAR in hDVA, vDVA and COP displacement between the normal and UVH or BVH groups. Table 2 shows the visual acuity and COP differences between the three groups before training. The results of the one-way ANOVA showed a significant difference in the LogMAR value in the horizontal dynamic visual acuity test (hDVALogMAR) (p = 0.002) and the LogMAR value in the vertical dynamic visual acuity test (vDVALogMAR) (p = 0.009). The post-hoc test showed a significant difference between normal and UVH in hDVALogMAR, but not between UVH and BVH. In vDVALogMAR, post-hoc tests showed significant difference between UVH and BVH, but not between normal and UVH. Simultaneous recording of COP while performing SVA and DVA tests showed no significant differences across groups. (ANOVA, p > 0.1) However, when we calculated the percentage difference of COP displacement in UVH and BVH patients while doing hDVA and vDVA (% diff. of COP = [UVH or BVH-normal/normal] × 100%), we found that BVH patients showed 41.92% and 48.97% more COP displacement than the normal group in hDVA and vDVA, respectively. The percentage differences in COP displacement between the UVH and normal groups were 21.15% in hDVA and 29.56% in vDVA, which were considerably less than those observed in BVH.

### 3.3. Effects of Rehabilitation in Visual Analog, DHI and ABC Scores

**Figure 3.**
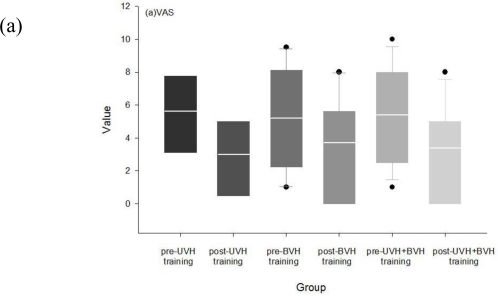

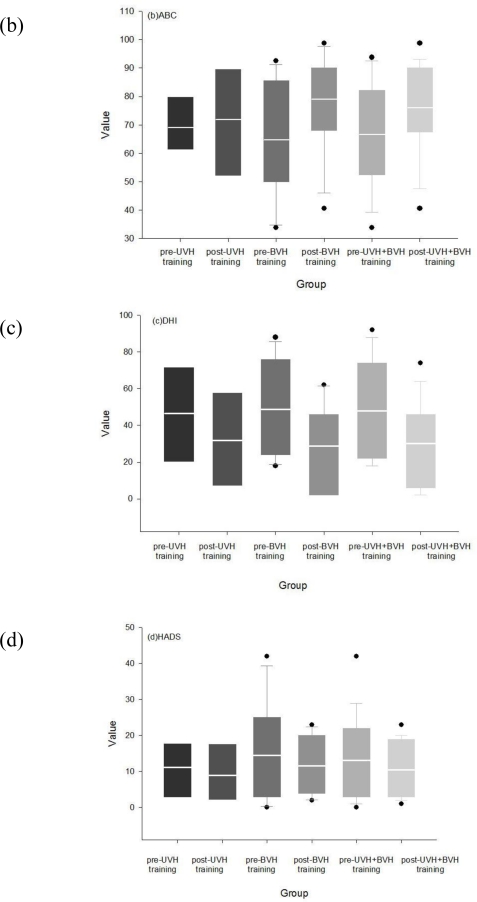
Change of subjective feeling using clinical assessment scales before and after vestibular training in UVH, BVH, and all patients. (a): VAS; (b): ABC; (c): DHI; (d): HADS.

Figure 3 illustrates the change of subjective scales before and after vestibular training. VAS scores improved significantly in all three groups (in UVH p = 0.042; in BVH p = 0.005; in UVH + BVH p = 0.001).

Significant improvements in ABC scores were found in the BVH group. DHI total score improved significantly in BVH and all patients. None of the three groups showed significant changes in HADS.

### 3.4. Effects of Rehabilitation on Dynamic Gait Index (DGI), Tinetti Scores and Timed “Up and Go” Test

Table 3 shows the change of objective scores before and after rehabilitation in UVH, BVH and all patients. Significant improvements were found for all parameters except for TUG in UVH and BVH patients.

### 3.5. Effects of Rehabilitation Training on DVA and COP

**Figure 4.**
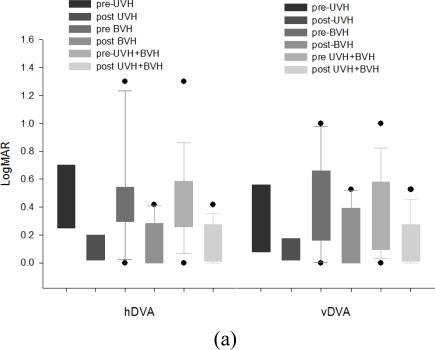

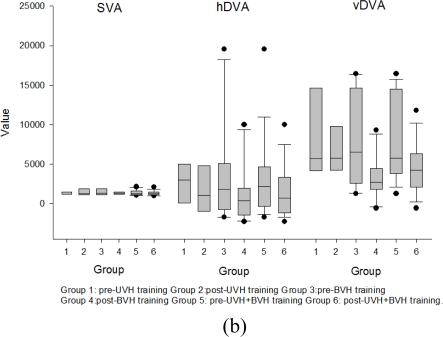
Change of visual acuity and COP displacement after rehabilitation exercises in UVH and BVH and all patients. (a): hDVA and vDVA scores in logMAR (b): COP during SVA, hDVA and vDVA.

Figure 4 shows the change of hDVA, vDVA, and COP displacement after 12 sessions (4.0 ± 1.22 weeks in UVH, 5.25 ± 1.39 weeks in BVH) of rehabilitation exercises in UVH and BVH patients. Both groups showed significant improvement in hDVA scores. (In UVH, p = 0.006, in BVH, p = 0.002, paired-*t* test). Significant improvement in vDVA scores was found in BVH (p = 0.002), but not in UVH patients. (p = 0.07). In UVH, COP displacement decreased by 14.68% (p = 0.036) during hDVA test, 15.75% (p = 0.192) during vDVA test. In BVH, COP displacement decreased by 30.69% (p = 0.162) during hDVA test, 37.83% (p = 0.011) during vDVA test.

### 3.6. Discussion

Our computerized system evaluated DVA and COP displacement simultaneously. The system was able to differentiate healthy individuals from vestibular patients. In this study, subjects with vestibular hypofunction demonstrated improvements in severity of dizziness, sense of confidence, balance and quality of life following vestibular rehabilitation. In addition to these subjective measures, there were significant improvements in dynamic visual acuity and COP displacement. Previous studies have shown similar improvements in visual acuity, oscillopsia and VOR gain in patients who underwent vestibular rehabilitation [4,33].

#### 3.6.1. Advantages of the Computerized Sensor System

The gyro and balance plate systems were triggered by subjects’ physical responses (*i.e.*, body sway and head rotation) for evaluation and training purposes. The investigator could read the signals acquired to the software and interpret subjects’ functional performance in real time. Results of each subject could be calculated immediately after each test trial, making this equipment an efficient assessment tool for clinical application. The head velocities of subjects could be transferred to the sensor system, which in turn would switch on the training scenes. Participants were motivated by immediate feedback from this computerized system, making rehabilitation exercises more entertaining.

#### 3.6.2. Differences of DVA and COP between Normal and Vestibular Deficits Groups before Rehabilitation

There was no significant difference of COP displacement in SVA test among three groups. The results indicated that the ability of static balance control in patients was the same as normal subjects. We found increments in the mean values of hDVALogMAR, vDVALogMAR and COP from normal to UVH to BVH groups. The differences reached a significant level in hDVA between normal and UVH, but not between UVH and BVH. In vDVA, significant differences were found between UVH and BVH, but not between normal and UVH. The differing results between UVH and BVH in hDVA and vDVA can be explained in several ways. Firstly, unlike other DVA tests, our DVA test was performed in a standing position. Most of our UVH and BVH patients reported more difficulty in moving their heads horizontally than vertically at a frequency >2 Hz while standing. This might be account for failure in differentiating UVH from BVH in our hDVA test. Secondly, our diagnosis was based on bithermal caloric test results. The caloric test is known to be a test primarily of the afferent neural pathway to the horizontal canals [34]. Vertical DVA detects gaze stability in the pitch plane and thus exhibits less sensitivity and specificity in comparison to the hDVA [35]. Lastly, the signal inputs are shared in the two ipsilateral vertical canals. Prior studies have shown that normal and UVH had similar vDVA scores [35], and vDVA test in the downward direction could differentiate normal subjects from those with UVH and BVH [36]. Our results were comparable with these findings. This may imply that the intact vertical canal functions can help patients maintaining gaze stability during active vertical head rotation.

#### 3.6.3. Improvements in Dynamic Visual Acuity & Balance Functions

After vestibular exercises involving repetitive head movements at 2 Hz, patients with vestibular hypofunction showed significant improvement in dynamic visual acuity. This finding indicates that visual acuity can be improved via vestibular rehabilitation exercises. Herdman *et al*. [37] identified similar results in patients with bilateral vestibular hypofunction. The same study further suggested that vestibular rehabilitative exercises were the only means by which one could significantly improve visual acuity during movement. Moreover, from earlier research with unilateral vestibular hypofunction patients, Herdman *et al*. [4] concluded that vestibular exercises could improve gaze stabilization during voluntary movements of the head. This result is consistent with the findings of the current study. For patients with vestibular hypofunction, vestibular rehabilitation is the main method by which dynamic visual acuity can be improved. Subjects by Herdman *et al*. who had received short periods of rehabilitation (less than 5 weeks in total) also improved their ability to maintain gaze stabilization [4]. In accordance with the results of this study, patients with vestibular hypofunction can expect improvement within a month of commencing specialized vestibular rehabilitation exercises.

In addition to improvements in visual acuity and COP, measurements of balance revealed that patients with vestibular hypofunction showed significant improvement after rehabilitation. In the DGI scores, patients improved from an initial average of 13.95 points to 19.47 points. Previous research has established a score <19 in DGI as a marker for higher fall risks in vestibular-deficits patients [38]. In this study, post rehabilitation DGI average scores in both UVH and BVH groups were greater than 19, indicating our rehabilitation exercises had beneficial effects on fall-risk reduction in UVH as well as BVH. Whitney *et al*. [38] pointed out that patients with vestibular hypofunction demonstrated similar improvements in DGI after rehabilitation [38,39]. Similar findings are reflected in this study. Following rehabilitation, Tinetti fall risk performance scales showed that patients transitioned from the category of “high fall risk” to “moderate fall risk”, while some even reached the “low fall risk” category. The average completion time of the TUG test prior to treatment was 11.15 seconds. Based on the cut-off point of 11.1 seconds, many of these patients were originally considered to have a high risk for falls. After rehabilitation, the average completion time reduced to 9.37 seconds. Again, these results indicate that subjects who underwent vestibular rehabilitation showed an improvement in dynamic visual acuity and subsequently whole body balance.

#### 3.6.4. Improvements in Quality of Life

Following vestibular rehabilitation in this study, patients recorded improvements in self-assessed quality of life measurements in all the dimensions including self-confidence, mood changes and severity of dizziness. Using the Activities-Specific Balance Confidence Scale (ABC), Powell and Myers [30] pointed out that results greater than 80 points represented a high level of function, while scores between 50 and 80 and less than 50 indicated a moderate and a low level of function, respectively. In this study, average confidence scores changed from 66.51 initially to 75.99 after rehabilitation. The change was more pronounced in BVH patients, possibly because BVH patients suffered more from the feeling of unsteadiness before training. Additionally, the average score increase of 9.48 indicates a fair improvement in confidence levels. The improvements may be explained by the fact that patients were experiencing less dizziness from head movements, and could thus execute daily activities with relative ease without having to worry about dizziness and imbalance. These findings were consistent with results from other investigations [11,38].

Dizziness Handicap Inventory (DHI) evaluations showed improvements in functional, emotional and physical abilities. While subject scores before and after rehabilitation both fell within the category for “moderate handicap”, there was a reduction from the initial score of 47.79 to 30.00, demonstrating a gradual transition from a moderate to a low degree of handicap. This was consistent with findings from Whitney *et al*. [38] and Meli *et al*. [11], who indicated that subjects experienced improvements in severity of dizziness. Score improvements of greater than 18 points in the DHI are defined as clinically significant [38]. In this study, the average score change was 17.79. These score increases are attributable, in part, to improvements in dizziness severity experienced by patients, which was reflected in a decreased handicap across categories of emotional, functional and physical performance.

Patients were also evaluated with the visual analog scale. Patients who had undergone vestibular rehabilitation improved on gaze stabilization and balance, thus leading to a decrease in dizziness severity. Presently, controversies exist between the relationship between visual acuity during movement and oscillopsia. Herdman *et al*. [37] indicated that no such relationship existed. Recently, Badaracco proposed that vertical DVA is correlated with oscillopsia score [36]. One possible explanation for this could be that studies assess visual acuity horizontally while oscillopsia is measured during vertical head movements in walking. In this study, we included both horizontal and vertical measurements of visual acuity. Furthermore, we also included both horizontal and vertical plane vestibular exercises. The improvements to oscillopsia could consequently have been due to patients receiving vestibular rehabilitation in the vertical plane. Alternatively, subjects may have had different levels of tolerance to retinal slip. Further research is required to determine the exact nature of the relationship between oscillopsia and DVA.

#### 3.6.5. Study Limitations

The first and most significant limitation of this study is the age of our control group. Previous studies have indicated that both hDVA and vDVA decreased with increasing age during active head movements [8,35]. Furthermore, decreased vestibular, visual and somatosensory responses in the elderly may cause inadequate responses to postural control, leading to an increase in postural sway [40–42]. Having difficulty recruiting older healthy volunteers, our control group mostly consisted of personnel from other laboratories and patients’ family members. Even though SVA and static COP were specifically controlled across the three groups, the significant younger age of the controls could still be confounding our DVA and COP assessments. This would affect the results of our pre-training group discrimination, which would make the new instrument less ideal for the differential diagnosis of normal subjects from UVH and BVH groups. The second limitation of this study was the diagnosis of our study patients groups. Apart from the classic test batteries for evaluation of dizzy patients such as ocular motility testing, positional/positioning testing, caloric testing, vestibular autorotation testing, growing amount of modern techniques including vestibular evoked myogenic potential (VEMP), whole body rotation testing, off-vertical rotation, subjective visual vertical and videooculography (VOG) provide more vestibular assessment strategies [43,44]. In our study, we used caloric test to discriminate between UVH, BVH and normal individuals. Despite the shortcomings of the caloric test, this test battery has been used for over half a century, and it is still considered the most sensitive test for detecting common vestibular abnormalities. The purpose of our study was to develop a system for evaluating the extent of functional improvements in patients with vestibular pathologies. Therefore, caloric test, with its sensitivity and specificity values of between 0.82 and 0.84 [45], is an assessment tool that fits the design of the present study. More work has to be done to incorporate the above-mentioned modern vestibular assessment strategies to improve diagnosis accuracy and comprehensiveness of functional outcome measurements. Another issue is the lack of non-exercising and conventional-exercising groups. Our intent was to investigate the rehabilitation effects of individuals with vestibular-deficits, not to answer questions about which group of patients would benefit more from our program. However, the study has provided sufficient evidence that patients with vestibular-deficits could, attain better outcomes from our computerized rehabilitation protocol.

## 4. Conclusions

Our computerized system efficiently evaluated and provided rehabilitation training to patients with vestibular deficits in our study group. This low price equipment with minimal environmental constraints may be applied to rehabilitation for vestibular patients and may help to improve their life quality.

## Figures and Tables

**Table 1.** Rehabilitation Protocol.

**Week**	**Standing positions**	**Vestibular exercises**
Week 1	Quiet stance	2 Hz horizontal head movement, naming the items on computer screen 2M in front
		2 Hz vertical head movement, naming the items on computer screen 2M in front
	Tandem stance	2 Hz horizontal head movement, naming the items on computer screen 2M in front
		2 Hz vertical head movement, naming the items on computer screen 2M in front
	Stepping	2 Hz horizontal head movement, naming the items on computer screen 2M in front
		2 Hz vertical head movement, naming the items on computer screen 2M in front

Week 2	Stepping	2 Hz horizontal head movement, naming the items on computer screen 2M in front
		2 Hz vertical head movement, naming the items on computer screen 2M in front
	Quiet stance	2 Hz horizontal head movement, naming revised items on computer screen 2M in front
		2 Hz vertical head movement, naming revised items on computer screen 2M in front
	Tandem stance	2 Hz horizontal head movement, naming revised items on computer screen 2M in front
		2 Hz vertical head movement, naming revised items on computer screen 2M in front

Week 3	Quiet stance on foam	2 Hz horizontal head movement, naming revised items on computer screen 2M in front
		2 Hz vertical head movement, naming revised items on computer screen 2M in front
	Tandem stance	2 Hz horizontal head movement, naming revised items on computer screen 2M in front
		2 Hz vertical head movement, naming revised items on computer screen 2M in front
	Stepping	2 Hz horizontal head movement, naming revised items on computer screen 2M in front
		2 Hz vertical head movement, naming revised items on computer screen 2M in front

Week 4	Quiet stance	2 Hz horizontal head movement, naming flashed items on computer screen 2M in front
		2 Hz vertical head movement, naming flashed items on computer screen 2M in front
	Quiet stance on foam	2 Hz horizontal head movement, naming flashed items on computer screen 2M in front
		2 Hz vertical head movement, naming flashed items on computer screen 2M in front
	Stepping	2 Hz horizontal head movement, naming flashed items on computer screen 2M in front
		2 Hz vertical head movement, naming flashed items on computer screen 2M in front

**Table 2.** Dynamic visual acuity and COP displacement differences between 3 groups before training. Data are shown in Mean ± Standard Error. ^#^significant difference among groups (p < 0.05); Letters in upper cases (A & B) next to the numbers indicates the results of post-hoc analysis. Same letters indicate no significant difference between the two groups, different letters indicate a significant difference between the two groups. COP in SVA/hDVA/vDVA: the displacement of center of pressure while performing static/horizontal dynamic/vertical dynamic visual acuity test. The unit of COP displacement is in millimeter (mm). UVH%diff. = [(UVH − Normal)/Normal] × 100%; BVH%diff. = [(BVH − Normal)/Normal] × 100%

	**hDVA LogMAR^#^**	**vDVA LogMAR^#^**	**COP in SVA (mm)**	**%diff**	**COP in hDVA (mm)**	**%diff**	**COP in vDVA (mm)**	**%diff**
Normal (N = 10)	0.08 ± 0.02^A^	0.09 ± 0.02 ^A^	1,230.27 ± 122.54	--	4,851.52 ± 1,185.78	--	7,383.43 ± 946.02	--
UVH (N = 20)	0.37 ± 0.08 ^B^	0.22 ± 0.07 ^A^	1,417.10 ± 128.61	15.19	5,877.85 ± 681.03	21.15	9,566.03 ± 1321.12	29.56
BVH (N = 21)	0.48 ± 0.09 ^B^	0.47 ± 0.10 ^B^	1,421.61 ± 114.53	15.55	6,885.46 ± 943.02	41.92	10,999.40 ± 1,055.77	48.97

**Table 3.** The change of clinical objective scores before and after rehabilitation in UVH, BVH and all patients. * indicates a significant difference before and after training. DGI: Dynamic gait index; Tinetti: Tinetti risk performance scale which includes balance and gait subscales, total scores = the sum of balance score and gait score; TUG: Timed “Up and Go” test in seconds. Data are shown in Mean ± Standard Error.

	**UVH (N = 20)**			**BVH (N = 21)**			**Vestibular-hypofunction (UVH + BVH) (N = 41)**		
	**before**	**after**	**p**	**before**	**after**	**p**	**before**	**after**	**p**
**DGI**	14.25 ± 0.88	19.5 ± 0.5	0.000*	13.73 ± 1.19	19.45 ± 0.82	0.000*	13.95 ± 0.77	19.47 ± 0.51	<0.001*
**Tinetti**									
balance	8 ± 0.65	12.25 ± 0.53	0.001*	8.82 ± 0.63	13.27 ± 0.62	0.000*	8.47 ± 0.45	12.84 ± 0.43	<0.001*
gait	8.88 ± 0.61	11 ± 0.19	0.004*	9.45 ± 0.47	11.09 ± 0.25	0.003*	9.21 ± 0.37	11.05 ± 0.16	<0.001*
total	16.88 ± 1.08	23.25 ± 0.45	0.001*	18.27 ± 1.03	24.36 ± 0.75	0.000*	17.68 ± 0.75	23.89 ± 0.48	<0.001*
**TUG**	11.58 ± 0.73	10.19 ± 0.72	0.051	10.85 ± 1.05	8.77 ± 0.68	0.01	11.15 ± 0.67	9.37 ± 0.51	<0.001*
